# Editorial: Health related quality of life inequalities

**DOI:** 10.3389/fpubh.2023.1214899

**Published:** 2023-05-26

**Authors:** John Yfantopoulos

**Affiliations:** IPOKE Research Institute, MBA National and Kapodistrian University of Athens, Athens, Greece

**Keywords:** EuroQol, health inequalities, Europe, globe, EuroQol 5 dimension (EQ-5D)

Health inequalities have been prevalent everywhere across the globe and throughout history. In Ancient Greek and Roman times, life expectancy was around 20 to 35 years (1). This estimate is based on “notoriously unrepresentative” graveyards and epitaphs of archons and no reference is made to the life span of slaves and the lower social classes. During the industrial revolution working class people lived shorter and unhealthier lives than the wealthier classes. Sir Edwin Chadwick, in his 1,842 English Sanitary Report (2), found that in Urban Liverpool the average age of death for professionals was 55 years, for farmers 22, and for mechanics/laborers it was only 15 years, (i.e., a staggering gap of almost 40 years) (2).

Circa 2000, the European Commission and the World Health Organization declared the reduction of health inequalities, between and within nations, as one of their prime objectives (3). Despite the substantial improvement in the health status of populations across the world, health inequalities between richer and poorer nations, as well as across different socioeconomic classes at each nation, have remained static or even, in some cases, widened (3).

Taking a global view, the 2022 World Health Report (4) demonstrates the existence of striking inequalities among nations (4). Life expectancy in the European Region is 78.2 years (75.1 males, 81.3 females) and in the African Region 64.5 years (62.4 males, 66.6 females). That is a health gap of 13.7 years (12.7 males, 14.7 females). Health inequalities are also apparent across all European Member States, regions and socio-economic groups (3). There is a 12 year gap in life expectancy for men across the EU (i.e., from 79.8 years in Italy to 68.0 in Lithuania), and an 8 year gap for women (i.e., from 85.3 in France to 77.4 in Bulgaria) (5).

Aristoteles in his Nicomachean Ethics (6), signifies the importance of equity as a moral issue and distinguishes between the “distributive justice” and the “corrective principle” of governmental intervention to reduce inequalities. Sir Marmot in a similar vein in a WHO Report, acknowledged that “health inequities are politically, socially and economically unacceptable (3, 5). They are also unfair, and the promotion of health equity is essential for the sustainable development of our European Health systems” (3, 5).

Documenting and reducing health inequalities constitutes a primary objective of public health policies. A large amount of research has been conducted among epidemiologists (7), economists (8), clinicians (8), sociologists (9), and public health experts (10), analyzing the psychological, gender, economic, demographic, and psychosocial aspects of health inequalities. Most research has mainly focused on aggregate objective indicators of health such as mortality, morbidity, and life expectancy. In the literature, research on patient reported outcomes such as HRQoL has become increasingly common as life expectancy increases and persons are living longer with chronic conditions. HRQoL is a useful global indicator to assess health inequalities between and within societies. The main goal of this Research Topic is to investigate the magnitude of inequalities in HRQoL using the EuroQol instrument. The research will analyze the differential and synergistic effects of a variety of characteristics that impact HRQoL across various countries globally. The research will make use of existing EuroQol surveys, launched in different countries.

Burström et al. examines the demographic composition and the social determinants of self-reported health for homeless people in Stockholm, Sweden initially in 2006 and later in 2018. They use the EQ-5D-5L instrument to assess the heath related quality of life of an extremely disadvantaged group with high rates of chronic illness and a highly deteriorated health status (EQ-VAS_2018_= 53.4).

Spronk et al. explore the magnitude of health inequalities in three selected European Countries. The EQ-5D-5L instrument was administered to a general population sample of 10,172 participants from Italy, the Netherlands, and the U.K. Chronic illness, and inability to work, were among the predominant factors contributing to health inequalities, followed by low educational levels (at a much smaller rate).

Tang et al. analyze the relationship between the economic burden of out-of-pocket payments for drugs, with the HRQoL for patients with chronic diseases, in five districts of China. A sample of 1,055 patients with chronic diseases were investigated and reported an overall average utility score of 0.727. Improvements in prevention, better access to drugs, and mainly reduction of out-of-pocket payments, would increase significantly the health-related quality of life of people with chronic diseases.

Szende et al. use the EQ-5D-3L instrument to assess health related quality of life inequalities for a sample of more than 100,000 participants in 18 countries across the globe. The estimated EQ-VAS concentration index varies from 0.090 to 0.157. In the decomposition analysis it was found that age and education were among the greatest contributors to health inequalities. From further analysis of seven countries with income data, it was found that the socio-economic variables of income and education were the most significant factors for health inequalities. In the 5 dimensions of the EQ-5D-3L descriptive system, usual activities and pain discomfort were the most significant contributors to health inequalities.

Tito et al. examine the relationship between HRQoL and treatment satisfaction of 357 cardiovascular patients in Ethiopia. Statistically significant negative correlations were found between HRQoL and the variables of unemployment, older age, previous hospitalization, non-adherence to lifestyle changes, and the presence of three or more cardiovascular diseases.

Yfantopoulos et al. focus on the measurement of HRQoL inequalities in Greece before and during the economic crisis. The EQ-5D-5L instrument was administered to a sample 4,177 young individuals in Greece. The economic crisis deteriorated HRQoL by 10.5% for the EQ-VAS and by 19.61% for the EQ-5D index. The health gap generated by the financial crisis was higher among the poor in comparison to the rich. Estimates of Theil inequality index before and during the crisis indicated an increase in income related HRQoL by 222.3% for the EQ-5D-5L index and by 124.2% for the EQ-VAS.

Reviewing the literature, it can be deduced that there has been limited use of quality-of-life measurements in health disparities. This special issue contributes to the dialogue of health inequalities by introducing the dimension of HRQoL. The aspiration is to raise awareness to the issue of health inequalities, by providing concrete evidence, which can later be utilized in shaping targeted and fair health policies. Investing in tackling health inequalities contributes to a more just, humane, and more equitable society with greater social cohesion and greater productivity.

## Author contributions

JY has prepared the manuscript draft, revised it for important intellectual content, contributed to the article, and approved the submitted version.
